# Health state utility values and associated complication-related difference in community-based adults with type 2 diabetes in Nanjing China: a cross-sectional study

**DOI:** 10.3389/fmed.2025.1599857

**Published:** 2025-08-26

**Authors:** Qiaoyu Huang, Hui Xie, Zhiyong Wang, Zhuanzhuan Fan, Simeng Sun, Ting Zhang, Huafeng Yang, Ya Shen

**Affiliations:** ^1^Department of Science and Education, Nanjing Municipal Center for Disease Control and Prevention, Nanjing, Jiangsu, China; ^2^The International Peace Maternity and Child Health Hospital, School of Medicine, Shanghai Jiao Tong University, Shanghai, China; ^3^Department of Basic Public Health, Nanjing Municipal Center for Disease Control and Prevention, Nanjing, Jiangsu, China; ^4^Department of Integrated Services, Jiangsu Provincial Center for Disease Control and Prevention, Nanjing, Jiangsu, China

**Keywords:** health utility, diabetes, community, adults, China

## Abstract

**Objectives:**

This study aimed to derive health state utility values (HSUVs) in community-based adults with type 2 diabetes from Nanjing, China, and to examine the differences associated with diabetes-related complications.

**Methods:**

A cross-sectional study employing a multi-stage random sampling method was conducted in Nanjing, China, in 2024. A total of 2,153 participants were finally included in the analysis. The Chinese version of EQ-5D-3L was used to assess health states, and the corresponding China value set was applied to convert these health states into HSUVs. Participants’ demographic characteristics and complication profiles were collected via a standardized questionnaire. Unadjusted and adjusted HSUV estimates were generated using multiple linear regression models with robust standard errors.

**Results:**

In the sample, 73.39% reported no problem in all five dimensions of the EQ-5D-3L, and none reported severe problems in any dimension. The mean overall HSUV was 0.9672. The unadjusted analyses showed that participants with any complication had lower HSUVs than those without any complication (all *p*-values < 0.001). In the fully adjusted model, participants with eye-related, cardiovascular, cerebrovascular and other complications (e.g., mental and oral diseases) were associated with decrements in HSUVs (all *p*-values < 0.05). Additionally, marital status and employment were identified as significant predictors of HSUVs (all *p*-values < 0.05).

**Conclusion:**

Our study suggests that health utility is not significantly impacted in community-based adults with type 2 diabetes in Nanjing, China. However, people with eye-related, cardiovascular and cerebrovascular complications may require additional attention and care to mitigate decrements in health utility.

## Introduction

Diabetes is widely acknowledged as a significant global public health concern. According to the Global Burden of Disease (GBD) Study, diabetes affected 529 million people worldwide in 2021 (1), while the Diabetes Atlas 2021 report by the International Diabetes Federation (IDF) estimates a similar figure of 537 million, corresponding to an age-standardized prevalence rate of 9.8% (2). The substantial epidemiological burden of diabetes is projected to escalate, with GBD forecasting 1.31 billion cases by 2050 and IDF forecasting 783 million cases (prevalence 11.2%) by 2045 (3).

The epidemiology of diabetes in China is particularly concerning. The IDF report reveals that approximately 140 million individuals aged 18–79 years were living with diabetes in 2021, resulting in a prevalence rate of 10.6% (4). This figure is projected to rise to 12.5% (4). Additionally, another projection study shows that the prevalence among Chinese adults would increase from 8.2% to 9.7% between 2020 and 2030 (5).

Diabetes is associated with a cascade of adverse health outcomes. The GBD study shows that diabetes accounted for 79.2 million disability-adjusted life years (DALYs) in 2021 globally (1). According to key facts released by the World Health Organization (WHO), diabetes remains a leading cause of blindness, kidney failure, heart attacks, stroke and lower limb amputation (6). Between 2000 and 2019, the mortality rate attributable to diabetes increased by 3% (6). A recent review highlights that although rates of cardiovascular complications and all-cause mortality among people with diabetes have declined in high-income countries, they have increased over time in low-to-middle-income countries (7). Furthermore, diabetes contributes significantly to premature mortality among individuals aged 30–70 years globally and accounts for a major proportion of deaths in low-income countries (8).

Diabetes also imposes a considerable economic and social burden. According to the WHO Global Burden Report on diabetes, the direct annual cost of diabetes worldwide exceeded USD 827 billion (9). Furthermore, the IDF estimates that most countries allocated 5%∼20% of total healthcare expenditures to diabetes treatment and management (10). Globally, healthcare expenditures for diabetes are projected to increase from USD 1.3 trillion in 2015 to over USD 2.0 trillion by 2030 (11). Another study reports that global spending on diabetes and its complications would rise to USD 802 billion by 2040 (12).

Given the substantial health, economic and social burden associated with diabetes, various interventions have been developed to address this issue with a long history (13). Although several interventions have demonstrated benefits to patients by improving clinical outcomes and their quality of life, it is important to note that the interventions may also carry potential risks, side effects or harms to other body functions (13, 14). Therefore, the pursuit of new and innovative interventions and optimization of existing strategies remain pivotal priorities in diabetes management and research.

Diabetes is closely related to lifestyle factors, necessitating robust community-based interventions for primary prevention and risk factor modification, as well as complication prevention among adults with diabetes. A meta-analysis, which included six trials involving 2,574 subjects, shows that community-based interventions significantly reduced fasting blood glucose and HbA1C levels among high-risk adults in low-to-middle income countries (15). Another global evidence synthesis demonstrates that community-based educational interventions decreased diabetes incidence by 54.0%, through mechanisms lowering fasting glucose, BMI, and waist circumference (16). Team-based care models – involving patients, their primary care providers, and one or two additional healthcare professionals (typically nurses or pharmacists) – have been shown to significantly decrease blood glucose levels and exhibit greater benefits in terms of blood pressure and lipid levels among adults with type 2 diabetes (17). Other evidence also shows similar results (18–20).

However, implementing community-based interventions in real-world settings requires not only evidence of effectiveness but also careful consideration of costs and cost-effectiveness, given the existence of multiple competing interventions and limited resources, particularly in resource-constrained settings or low-income countries. Since interventions vary in terms of their designs, administration modes, frequency, target populations, and outcome measurements, identifying the most cost-effective intervention remains challenging. Therefore, it is crucial to establish standardized outcome measures or comparability of effectiveness metrics across competing interventions for the assessment of cost-effectiveness of interventions (21).

The quality-adjusted life year (QALY) has been widely adopted as a comparable outcome measure in health economic evaluations. This measure integrates both the length and health-related quality of life, allowing for characterizing intervention effects on a universal and comparable scale (22). An essential step in QALY calculation involves eliciting and applying health-related quality of life weights, also known as health state utility values (HSUVs). Health state utility is a preference-based measure that assesses the desirability or preference for different health states, typically ranging from 0 (indicating death) to 1 (representing perfect or optimal health) (23).

Previous studies have evaluated HSUVs among adults with diabetes in Chinese settings, but they were limited to the exploration of one single complication of diabetes (24), only hospital settings (25, 26), or failure to specify the tariff they referred to for the derivation of HSUVs from multi-attribute utility instruments (25, 27). To contribute to this field, the current study aimed to elicit HSUVs among community-based adults with type 2 diabetes in Nanjing, China and examine complication-related difference.

## Materials and methods

### Study design and sample

This is a cross-sectional study based on data from a field survey of economic burden in community-based adults with type 2 diabetes in Nanjing, China. The survey was conducted from June to August 2024. Ethical review and approval for this survey were obtained from the institutional review board of the Nanjing Center for Disease Control and Prevention (PJ2022-A001-02).

A multi-stage random sampling method was used for the selection of participants. First, three sub-districts were randomly selected from each of the 11 districts of Nanjing. Second, one primary health care center was randomly selected from each of the selected sub-districts. Third, 100 participants were randomly targeted and invited to participate in the survey from the diabetes registry pool of each selected primary health care center. Participants were surveyed if they: (1) were diagnosed with type 2 diabetes; (2) had diabetes for no less than six months; (3) were no less than 18 years old; (4) provided oral consent to participate in the survey. A total of 3,129 participants completed the survey. After excluding any missing values associated with the analyzed variables, a complete-case sample of 2,153 participants was determined for analyses.

### Health utility

The Chinese version of EQ-5D-3L was employed to elicit HSUVs. Participants completed the EQ-5D-3L in a self-administration mode. During the administration, participants were instructed to answer how they felt in terms of the five dimensions: mobility, self-care, daily activity, pain/discomfort, and anxiety/depression. Responses included three levels corresponding to no problems, some problems and severe problems. Consequently, each participant’s health can be described by the five dimensions with each rated at a specific level. For example, a “11111” description means perfect health as there is no problems reported on any dimension; a “33333” description indicates the worst health as there is a severe problem reported in any dimension. Using the China value set for EQ-5D-3L (28), the health state descriptions can be converted into HSUVs, with 1 indicating the best health (corresponding to “11111” state) and 0.1702 the worst health (corresponding to “33333” state).

### Variables

Diabetes-related information was collected using self-administered questionnaires. Participants were asked about how long they had suffered from diabetes, the complications they had, current treatments and blood glucose control.

Demographic and socioeconomic variables included sex, age, marital status, education, employment, registered residence (Hu Kou) and medical insurance coverage. Health behavior-related variables included height, weight, current smoking status, current drinking status, and physical activity.

### Statistical analysis

Mean (standard deviation, SD as an abbreviation) and frequency (percentage) were used to describe sample characteristics. Crude mean (SD) and 95% confidence interval (CI) were computed to present the distribution of HSUVs, overall and by complication- and treatment-related groups. The 95% CI and significance of the crude difference in HSUVs between groups were estimated using crude linear regressions with robust standard errors.

To examine the adjusted complication-related difference in HSUVs, a set of linear regression models was developed. First, a crude model was fitted with only complication variables as predictors (Crude model). Second, based on the crude model, sex, age, diabetic duration, marital status, education, registered residence, medical insurance coverage and employment were additionally included as controlled variables in the model (Model 1). Third, based on Model 1, current smoking status, current drinking status, physical activity, body mass index and family history of diabetes were additionally included as controlled variables in the model (Model 2). Fourth, based on Model 2, glucose control status, diet treatment, exercise treatment, oral medication treatment and insulin treatment were additionally included as controlled variables in the model (Model 3). The coefficients of the complication variables and associated changes across the four models were of interest. All linear regression models were fitted with robust standard errors, as recommended by Devlin et al. and other similar HSUV studies (29, 30). Multicollinearity was assessed using the variance inflation factor. Data analyses were performed in R version 4.4.1. Statistical significance was indicated by a *p*-value less than 0.05.

## Results

### Sample characteristics and prevalence of diabetic complications

In the sample, both males and females accounted for almost half (49.65% vs. 50.35%). The mean age was 59.31(SD = 10.80) years. Nearly all were married (91.73%). Around one third completed junior high school education (28.89%) while over one fifth dropped out of elementary school or were illiterate (20.95%). Over one third were currently employed (36.41%). The mean number of complications was 0.52 (SD = 1.18). The most common complication was eye-related (11.94%), followed by nephropathy-related (9.20%) and peripheral neuropathy-related (8.41%). The mean diabetic duration was 7.77 (SD = 6.96) years. Further details are provided in Table 1.

**TABLE 1:** Descriptive statistics for socio-economic and diabetes-related characteristics (*N* = 2,153).

Variables	*N* (%)
**Sex**	
Male	1,069 (49.65)
Female	1,084 (50.35)
**Age, mean (SD)**	59.31 (10.80)
Adults (< 45 years)	150 (6.97)
Middle-aged (45∼59 years)	1,084 (50.35)
Older (≥ 60 years)	919 (42.68)
**Marital status**	
Single	43 (2.00)
Married	1,975 (91.73)
Divorced/separated/widowed	135 (6.27)
**Education**	
Below elementary school	451 (20.95)
Elementary school	449 (20.85)
Junior high school	622 (28.89)
High school	348 (16.16)
College	283 (13.14)
**Hukou/registered residence**	
Rural	1,331 (61.82)
Urban	822 (38.18)
**Medical insurance coverage**	
City employees’ basic medical insurance	833 (38.69)
City residents’ basic medical insurance	1,288 (59.82)
No coverage	32 (1.49)
**Employment**	
Employed	784 (36.41)
Retired	601 (27.91)
Unemployed	768 (35.67)
**Diabetic duration**	7.77 (6.96)
**Family diabetes history**	
No	1,355 (62.94)
Yes	798 (37.06)
**Nephropathy-related complications**	
No	1,955 (90.80)
Yes	198 (9.20)
**Eye-related complications**	
No	1,896 (88.06)
Yes	257 (11.94)
**Foot-related complications**	
No	2,012 (93.45)
Yes	141 (6.55)
**Cardiovascular complications**	
No	2,004 (93.08)
Yes	149 (6.92)
**Cerebrovascular complications**	
No	2,011 (93.40)
Yes	142 (6.60)
**Peripheral neuropathy-related complications**	
No	1,972 (91.59)
Yes	181 (8.41)
**Other complications**	
No	2,101 (97.58)
Yes	52 (2.42)
**Number of complications**	
0	1,598 (74.22)
1	312 (14.49)
2 and more	243 (11.29)
**Health state utility values, mean (SD)**	0.9672 (0.0776)

SD, standard deviation.

### Descriptive statistics for HSUVs overall and by groups

Approximately three fourths of the participants (73.39%) reported no problems on all five dimensions of the EQ-5D-3L, denoted as a health state of “11111”. No participant reported severe problems on the five dimensions, denoted as a “33333” health state. The overall mean HSUV was 0.9672 (SD = 0.0776).

The crude mean, SD and 95% CI for HSUVs were presented for each group of complications and treatments in Tables 2, 3. All groups except the cerebrovascular group (mean HSUV = 0.8446) had a mean HSUV above 0.85. A significant difference in crude HSUVs was observed between among complication groups (all *p*-values < 0.001). In general, participants with complications had lower HSUVs than those without.

**TABLE 2:** Descriptive statistics for health state utility values by complication-related groups (*N* = 2,153).

Complication groups	Crude mean (SD)	95% CI*	*P*-value
**Nephropathy-related**			
No	0.9733 (0.0692)	0.9703, 0.9764	< 0.001
Yes	0.9072 (0.1192)	0.8906, 0.9238	–
**Eye-related**			
No	0.9822 (0.0549)	0.9797, 0.9847	< 0.001
Yes	0.8570 (0.1201)	0.8424, 0.8717	–
**Foot-related**			
No	0.9747 (0.0673)	0.9718, 0.9776	< 0.001
Yes	0.8608 (0.1237)	0.8405, 0.8812	–
**Cardiovascular**			
No	0.9754 (0.0667)	0.9725, 0.9783	< 0.001
Yes	0.8576 (0.1201)	0.8384, 0.8768	–
**Cerebrovascular**			
No	0.9759 (0.0651)	0.9731, 0.9787	< 0.001
Yes	0.8446 (0.1234)	0.8244, 0.8649	–
**Peripheral neuropathy-related**			
No	0.9731 (0.0711)	0.9699, 0.9762	< 0.001
Yes	0.9036 (0.1103)	0.8876, 0.9196	–
**Other**			
No	0.9691 (0.0759)	0.9659, 0.9724	< 0.001
Yes	0.8916 (0.1036)	0.8637, 0.9195	–
**Number of complications**			
0	0.9936 (0.0265)	0.9924, 0.9949	–
1	0.9145 (0.1099)	0.9023, 0.9267	< 0.001
2 and more	0.8613 (0.1181)	0.8465, 0.8762	< 0.001

*95% CIs were estimated with crude linear regression models with robust standard errors. SD, standard deviation; CI, confidence interval.

**TABLE 3:** Descriptive statistics for health state utility values by treatment-related groups (*N* = 2,153).

Treatment	Crude mean (SD)	95% CI*	*P*-value
**Diet treatment**			
No	0.9676 (0.0800)	0.9609, 0.9743	0.901
Yes	0.9671 (0.0768)	0.9634, 0.9709	–
**Exercise treatment**			
No	0.9641 (0.0802)	0.9591, 0.9691	0.080
Yes	0.9700 (0.0751)	0.9656, 0.9743	–
**Oral medication treatment**			
No	0.9558 (0.0885)	0.9491, 0.9625	< 0.001
Yes	0.9725 (0.0715)	0.9688, 0.9761	–
**Insulin treatment**			
No	0.9677 (0.0779)	0.9643, 0.9711	0.152
Yes	0.9574 (0.0701)	0.9438, 0.9711	–

*95% CIs were estimated with crude linear regression models with robust standard errors. SD, standard deviation; CI, confidence interval.

When analyzing complication counts, those with one or more complications had significantly lower HSUVs compared to complication-free participants (all *p*-values < 0.001). Participants receiving oral medication treatment demonstrated higher HSUVs than those not receiving such treatments (*p* < 0.001).

### Adjusted difference in HSUVs between complication groups

The full model results can be found in the Supplementary appendix. The main results can be found in Table 4. Participants who had eye-related complications were associated with lower HSUVs (coef. = −0.0939, *p* < 0.001 from Model 3, the fully adjusted model). The magnitude, direction and significance of this effect remained nearly consistent across the four models (all *p*-values < 0.001). Similar patterns were observed for the difference in HSUVs between cardiovascular, cerebrovascular and other complications (e.g., mental and oral conditions) groups.

**TABLE 4:** Regression results for complication-related factors associated with health state utility values (*N* = 2,153).

Variable	Crude model*		Model 1*		Model 2*		Model 3*	
	Coef. (95% CI)	*P*-value	Coef. (95% CI)	*P*-value	Coef. (95% CI)	*P*-value	Coef. (95% CI)	*P*-value
**Nephropathy-related**								
No	Ref.		Ref.		Ref.		Ref.	–
Yes	0.0154 (−0.0003∼0.0311)	0.054	0.0143 (−0.0013∼0.0299)	0.072	0.0148 (−0.0007∼0.0303)	0.061	0.0153 (−0.0002∼0.0308)	0.054
**Eye-related**								
No	Ref.		Ref.		Ref.		Ref.	–
Yes	−0.0982 (−0.1167∼−0.0797)	< 0.001	−0.0964 (−0.1151∼−0.0777)	< 0.001	−0.0964 (−0.1151∼−0.0777)	< 0.001	−0.0939 (−0.1125∼−0.0753)	< 0.001
**Foot-related**								
No	Ref.		Ref.		Ref.		Ref.	–
Yes	0.0027 (−0.0294∼0.0347)	0.869	0.0029 (−0.0290∼0.0349)	0.857	0.0025 (−0.0293∼0.0343)	0.879	−0.0031 (−0.0345∼0.0284)	0.848
**Cardiovascular**								
No	Ref.		Ref.		Ref.		Ref.	–
Yes	−0.0328 (−0.0626∼−0.0030)	0.031	−0.0315 (−0.0606∼−0.0025)	0.034	−0.0306 (−0.0597∼−0.0016)	0.038	−0.0313 (−0.0598∼−0.0028)	0.031
**Cerebrovascular**								
No	Ref.		Ref.		Ref.		Ref.	–
Yes	−0.0755 (−0.1027∼−0.0483)	< 0.001	−0.0739 (−0.1008∼−0.0471)	< 0.001	−0.0737 (−0.1005∼−0.0468)	< 0.001	−0.0730 (−0.0996∼−0.0463)	< 0.001
**Peripheral neuropathy-related**								
No	Ref.		Ref.		Ref.		Ref.	–
Yes	−0.0037 (−0.0191∼0.0117)	0.639	−0.0019 (−0.0169∼0.0132)	0.809	−0.0017 (−0.0166∼0.0132)	0.820	−0.0010 (−0.0158∼0.0137)	0.891
**Other complications**								
No	Ref.		Ref.		Ref.		Ref.	–
Yes	−0.0460 (−0.0721∼−0.0200)	0.001	−0.0480 (−0.0736∼−0.0223)	< 0.001	−0.0472 (−0.0729∼−0.0216)	< 0.001	−0.0453 (−0.0709∼−0.0197)	< 0.001

*The crude model: regressing utility values on all complication groups only. The Model 1: independent variables in the crude model plus sex, age, diabetic duration, marital status, education, hukou, medical insurance coverage and employment. The model 2: independent variables in the model 1 plus current smoking status, current drinking status, physical activity, body mass index, family history of diabetes. The model 3: independent variables in the model 2 plus glucose control status, diet treatment, exercise treatment, oral medication treatment and insulin treatment. Models were fitted with robust standard errors. coef., coefficient; CI, confidence interval; ref., reference group.

In Model 3, marital status and employment were also identified as significant predictors of HSUVs. Specifically, compared with participants who were single, those who were married had higher HSUVs (coef. = 0.0325, *p* = 0.028). Compared with participants who were employed, those who were not had lower HSUVs (coef. = −0.0104, *p* = 0.004).

## Discussion

Using a random community-based sample of adults with type 2 diabetes from Nanjing, China, this study explored HSUVs and differences related to diabetic complications in this population. The study found an overall HSUV of 0.9672, and showed significant differences in HSUVs among eye-related, cardiovascular, cerebrovascular, and other complications groups (e.g., mental and oral diseases). As a commonly applied health outcome measure, HSUVs and resulting QALYs enable comparisons between interventions or programs that have various features. Investigating HSUVs has been widely carried out in different populations across different locations.

Pan investigated HSUVs using EQ-5D-5L among a cohort of community-dwelling people with type 2 diabetes and eye-related complications (24). They reported a mean HSUV of 0.983 (SD = 0.067), which is similar to the results of our study. The similarity in results may be attributable to similar utility elicitation instruments (despite different versions) (31) and comparable study settings. Particularly, Pan’s study was conducted in Suzhou whereas our study was conducted in Nanjing. Both cities are comparable in terms of socioeconomic levels. Zhang examined HSUVs using the 15D instrument in community-based people with type 2 diabetes in Qingdao, China and reported mean HSUVs of 0.971, 0.972, and 0.960 for diabetes-free, newly detected diabetes through screening and previously known diabetes, respectively (27). Our study found similar results when compared with Zhang’s study. Despite the use of different elicitation instruments (EQ-5D vs. 15D), it appeared that this did not have substantial impacts on HSUVs, as demonstrated by a previous study (31).

Wang et al.’s Hong Kong study using the SF-6D instrument reported a mean HSUV of 0.868 among people with type 2 diabetes in primary care settings (32). Another Hong Kong study employing the same instrument found a mean HSUV of 0.882 among people with type 2 diabetes without complications managed in primary and secondary care settings (30). In our study, we used EQ-5D-3L to elicit HSUVs among individuals with type 2 diabetes managed in primary care setting. We found that our results were higher than those elicited via SF-6D in the two Hong Kong studies.

It is acknowledged that HSUVs, or health preferences, can differ across countries, regions and locations with diverse social and cultural features (33, 34). This may serve as one explanation for the difference observed. In addition to study settings, the elicitation methods or instruments used may also play crucial roles in shaping the difference in HSUVs. Currently, HSUVs can be elicited using direct or indirect methods (22). Indirect methods generally involve using multi-attribute utility instruments, including EQ-5D, SF-6D, and HUI-2/HUI-3 (22). Previous studies demonstrated that HSUVs elicited with the SF-6D were higher than those elicited with the EQ-5D (35, 36). The difference between EQ-5D and HUI-3 was also investigated and it was found that HSUVs elicited with the EQ-5D were higher than those elicited with the HUI-3 (31). The higher HSUVs elicited using the EQ-5D may be attributable to fewer dimensions and lower sensitivity compared to other instruments (36).

Our study reported an overall HSUV of 0.9672 in the studied population, which was slightly lower than the Chinese norms of 0.985 for the EQ-5D-3L (37). As to the observed tiny difference, we might cite the following explanations. First, the participants in our study were community-based, indicating that the severity of diabetes would be relatively mild and their daily functions would be well-preserved. As the EQ-5D is a generic instrument for assessing health preference, it may not be highly sensitive to the impact imposed by diabetes or diabetes-related complications (38, 39). Second, residing in communities or managed by primary care, people with type 2 diabetes are more likely to be exposed to and receive effective community-based interventions that prevent, manage and delay the disease (20, 40, 41).

Diabetes is often accompanied by various complications (42–44). The complications could impose a significant disease burden at the societal level and cause adverse health outcomes at the individual level (45). Our study demonstrated that eye-related, cardiovascular, cerebrovascular and other complications (e.g., mental and oral diseases) were associated with a decrement in HSUVs. This result was consistent with that reported in a systematic review focused on HSUVs in type 2 diabetes in East and Southeast Asia (46), and that reported in a systematic review focused on the global population of type 2 diabetes (47). However, we did not observe significant decrements in HSUVs for foot-related, peripheral neuropathy-related and nephropathy-related complications. We might provide the following explanations. First, we did not collect data on the severity of complications, which limited our ability to examine the effects of severity on HSUVs. For example, foot-related complications could potentially have effects on HSUVs due to their impact on mobility and daily activity, as measured by two dimensions in the EQ-5D instrument. If participants had severe diabetic feet, they might have been sensitive to the two dimensions, leading to variations in HSUVs. Second, we speculated that the complications studied in our study could interplay concerning their physiological mechanisms. This could mask the effects of one complication by another, making it difficult to detect significant decrements in HSUVs for certain complications.

Our study may have significant implications for research and policy. First, as community-based interventions gain increasing importance in the management of type 2 diabetes, evaluation and selection of cost-effective interventions are of high urgency. The HSUV estimates obtained in our study can benefit these processes by informing the parameters of economic models. Additionally, the determination of the complication-related profile of HSUVs can facilitate an evaluation that considers full information on the outcomes associated with complications. Second, our study might contribute to the understanding of the use of the EQ-5D instrument among community-based individuals with type 2 diabetes. By providing a set of HSUVs as a reference and discussing the potential ceiling effects of this instrument in this population, we can add to the existing knowledge.

Limitations of our study should be noted. First, although self-report administration mode is allowed in the use of the EQ-5D, it may cause recall bias or random responses. We want to point out that the insignificant effects of some complications may be attributable to this bias. Second, we failed to include participants living in rural areas, which could limit the generalizability of our study results. A previous study revealed that HSUVs exhibited substantial variation between participants living in urban and rural areas (48). Therefore, future studies are warranted to include a more diverse sample of participants to address this limitation.

## Conclusion

Our study suggests that health utility is not significantly impacted in community-based adults with type 2 diabetes in Nanjing, China. However, people with eye-related, cardiovascular and cerebrovascular complications may require additional attention and care to mitigate decrements in health utility.

## Data Availability

The raw data supporting the conclusions of this article will be made available by the authors, without undue reservation.
