# Supplementary material for: Health state utility values and associated complication-related difference in community-based adults with type 2 diabetes in Nanjing China: a cross-sectional study
Source: Front Med (Lausanne). 2025 Aug 26;12:1599857. doi: 10.3389/fmed.2025.1599857 (PMC12417432; doi:10.3389/fmed.2025.1599857)
Supplement: Supplementary file 1 [file Data_Sheet_1.pdf]

Appendix to the work "Health state utility values and associated complication-related difference in community-based adults with type 2 diabetes in Nanjing China: a cross-sectional study"

Table 1 Crude Model Results

Variables	Coefficient	CI-lower limit	CI-upper limit	P value
Nephropathy-related	0.01541	-0.00029	0.03111	0.05434
Eye-related	-0.09818	-0.11668	-0.07968	0
Foot-related	0.00269	-0.02935	0.03473	0.86931
Cardiovascular	-0.03276	-0.06256	-0.00296	0.03121
Cerebrovascular	-0.07551	-0.10272	-0.0483	0
Peripheral neuropathy-related	-0.00368	-0.01906	0.0117	0.6391
Other	-0.04603	-0.07207	-0.01999	0.00054

Table 2 Model 1 Results

Variables	Coefficient	CI-lower limit	CI-upper limit	P value
Nephropathy-related	0.01431	-0.00127	0.02989	0.07183
Eye-related	-0.09643	-0.11512	-0.07774	0
Foot-related	0.00293	-0.02899	0.03486	0.85708
Cardiovascular	-0.03154	-0.06064	-0.00245	0.0336
Cerebrovascular	-0.07394	-0.1008	-0.04708	0
Peripheral neuropathy-related	-0.00185	-0.01686	0.01316	0.80899

Other	-0.04796	-0.07363	-0.0223	0.00025
Sex_female	0.00098	-0.00447	0.00642	0.72488
Age	-0.00028	-0.00067	0.00011	0.15548
Diabetic duration	-0.00027	-8.00E-04	0.00027	0.32432
Marriage_married	0.0347	0.00512	0.06429	0.02152
Marriage_ divorced/separated/widowed	0.02412	-0.00767	0.05591	0.13699
Education_elementary school	0.00407	-0.00432	0.01246	0.34141
Education_junior high school	6.00E-04	-0.00802	0.00923	0.89129
Education_high school	-0.00516	-0.0169	0.00659	0.38906
Education_colleage	-0.00114	-0.01322	0.01094	0.85367
Hukou/registered residence_urban	0.00134	-0.00635	0.00903	0.73312
Medical insurance coverage_ city residents' basic medical insurance	0.00184	-0.00397	0.00765	0.53492
Medical insurance coverage_ no coverage	-0.01422	-0.05356	0.02512	0.47858
Employment_ retired	-0.00355	-0.01271	0.00562	0.44791
Employment_ unemployed	-0.0102	-0.01729	-0.00311	0.00482

Table 3 Model 2 Results

**Variables**	**Coefficient**	**CI-lower limit**	**CI-upper limit**	**P value**
Nephropathy-related	0.0148	-0.00068	0.03028	0.06102
Eye-related	-0.09641	-0.11511	-0.07771	0
Foot-related	0.00247	-0.02934	0.03429	0.87885

Cardiovascular	-0.03064	-0.05966	-0.00163	0.03848
Cerebrovascular	-0.07367	-0.10054	-0.0468	0
Peripheral neuropathy-related	-0.00173	-0.01664	0.01319	0.82047
Other	-0.04722	-0.07288	-0.02157	0.00031
Sex_female	0.00249	-0.00404	0.00903	0.4547
Age	-0.00032	-0.00071	8.00E-05	0.11842
Diabetic duration	-0.00021	-0.00075	0.00034	0.45551
Marriage_married	0.03387	0.00403	0.0637	0.0261
Marriage_ divorced/separated/widowed	0.02301	-0.00899	0.05501	0.15864
Education_elementary school	0.00358	-0.0047	0.01186	0.39685
Education_junior high school	-3.00E-04	-0.00888	0.00828	0.9461
Education_high school	-0.00636	-0.01797	0.00525	0.28267
Education_colleage	-0.00225	-0.01433	0.00983	0.7149
Hukou/registered residence_urban	0.00163	-0.00613	0.0094	0.67993
Medical insurance coverage_ city residents’ basic medical insurance	0.00174	-0.0041	0.00758	0.55946
Medical insurance coverage_ no coverage	-0.01415	-0.05345	0.02515	0.48029
Employment_ retired	-0.00478	-0.01406	0.00449	0.31172
Employment_ unemployed	-0.0109	-0.01803	-0.00378	0.00272
Current smoking_yes	-0.00035	-0.00781	0.00711	0.92658
Current drinking_yes	0.00391	-0.00425	0.01206	0.34771
Physical activity_yes	0.00615	0.00071	0.01159	0.02672
Body mass index	1.00E-04	-0.00073	0.00092	0.81865
Diabetes family history_yes	-0.00369	-0.00954	0.00216	0.21642

Table 4 Model 3 Results

Variables	Coefficient	CI-lower limit	CI-upper limit	P value
Nephropathy-related	0.01527	-0.00024	0.03078	0.05363
Eye-related	-0.09391	-0.11251	-0.0753	0
Foot-related	-0.00307	-0.0345	0.02836	0.84802
Cardiovascular	-0.03128	-0.05975	-0.00282	0.03127
Cerebrovascular	-0.07296	-0.09963	-0.04629	0
Peripheral neuropathy-related	-0.00103	-0.01576	0.0137	0.89068
Other	-0.04532	-0.07094	-0.0197	0.00053
Sex_female	0.00261	-0.00397	0.00919	0.43696
Age	-0.00037	-0.00076	2.00E-05	0.06616
Diabetic duration	-8.00E-05	-0.00062	0.00045	0.7575
Marriage_married	0.03248	0.00352	0.06145	0.02796
Marriage_ divorced/separated/widowed	0.0221	-0.00903	0.05324	0.16404
Education_elementary school	0.00396	-0.00428	0.0122	0.34571
Education_junior high school	-0.00047	-0.00901	0.00808	0.91462
Education_high school	-0.00526	-0.01676	0.00625	0.3705
Education_colleage	0.00036	-0.01184	0.01255	0.9542
Hukou/registered residence_urban	0.00162	-0.00608	0.00933	0.67938
Medical insurance coverage_ city residents' basic medical insurance	0.0016	-0.00421	0.00742	0.58859
Medical insurance coverage_ no coverage	-0.01632	-0.05536	0.02271	0.41219
Employment_ retired	-0.00475	-0.01395	0.00445	0.31106
Employment_ unemployed	-0.01041	-0.0175	-0.00331	0.00405
Current smoking_yes	0.00133	-0.00593	0.00858	0.7198
Current drinking_yes	0.00373	-0.00441	0.01187	0.3691

Physical activity_yes	0.00243	-0.00364	0.0085	0.43174
Body mass index	0.00022	-0.00061	0.00104	0.60782
Diabetes family history_yes	-0.00428	-0.01012	0.00155	0.15015
Glucose control_yes	0.00469	-0.00265	0.01204	0.21044
Glucose control_unknown	-0.0144	-0.03522	0.00641	0.17491
Diet treatment_yes	0.00121	-0.00613	0.00856	0.74599
Exercise treatment_yes	0.00917	0.0024	0.01593	0.00798
Oral medication treatment_yes	0.01297	0.00613	0.01981	0.00021
Insulin treatment_yes	0.01357	0.00152	0.02562	0.02727
